# AGS3-based optogenetic GDI induces GPCR-independent Gβγ signalling and macrophage migration

**DOI:** 10.1098/rsob.240181

**Published:** 2025-02-05

**Authors:** Waruna Thotamune, Sithurandi Ubeysinghe, Chathuri Rajarathna, Dinesh Kankanamge, Koshala Olupothage, Aditya Chandu, Bryan A. Copits, Ajith Karunarathne

**Affiliations:** ^1^ Department of Chemistry, Saint Louis University, Saint Louis, MO 63103, USA; ^2^ Institute for Drug and Biotherapeutic Innovation, Saint Louis University, Saint Louis, MO 63103, USA; ^3^ Department of Anesthesiology, Washington University Pain Center, Washington University School of Medicine, St. Louis, MO 63110, USA; ^4^ Department of Chemistry and Biochemistry, The University of Toledo, Toledo, OH 43606, USA

**Keywords:** GPCR, G proteins, GDI, optogenetics, macrophage migration, activators of G-protein signalling 3

## Abstract

G-protein-coupled receptors (GPCRs) are efficient guanine nucleotide exchange factors (GEFs) and exchange GDP to GTP on the Gα subunit of G-protein heterotrimers in response to various extracellular stimuli, including neurotransmitters and light. GPCRs primarily broadcast signals through activated G proteins, GαGTP and free Gβγ and are major disease drivers. Evidence shows that the ambient low threshold signalling required for cells is likely supplemented by signalling regulators such as non-GPCR GEFs and guanine nucleotide dissociation inhibitors (GDIs). Activators of G-protein signalling 3 (AGS3) are recognized as a GDI involved in multiple health and disease-related processes. Nevertheless, understanding of AGS3 is limited, and no significant information is available on its structure–function relationship or signalling regulation in living cells. Here, we employed *in silico* structure-guided engineering of a novel optogenetic GDI, based on the AGS3’s G-protein regulatory motif, to understand its GDI activity and induce standalone Gβγ signalling in living cells on optical command. Our results demonstrate that plasma membrane recruitment of OptoGDI efficiently releases Gβγ, and its subcellular targeting generated localized PIP3 and triggered macrophage migration. Therefore, we propose OptoGDI as a powerful tool for optically dissecting GDI-mediated signalling pathways and triggering GPCR-independent Gβγ signalling in cells and *in vivo*.

**Figure:**
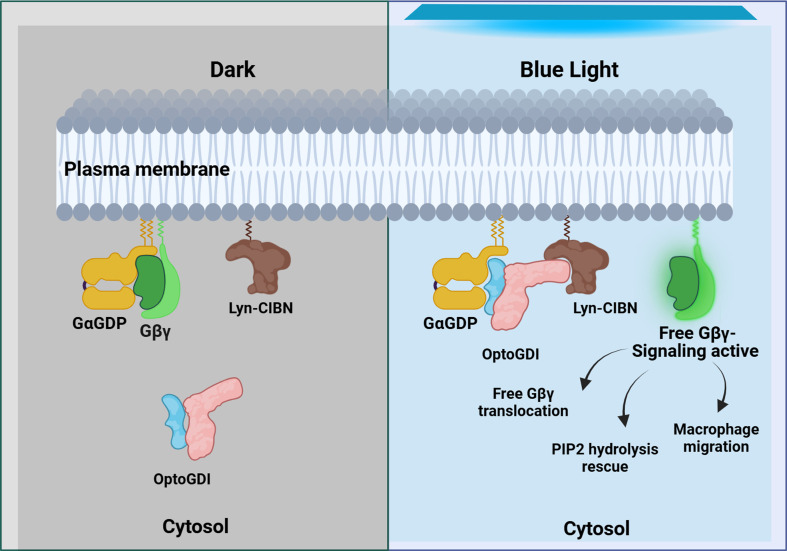

## 1. Introduction

G-protein-coupled receptors (GPCRs) represent approximately 5% of the human genome, have immense pathophysiological significance and are thus a primary target for drug discovery [1–4]. GPCRs constitute one of the largest and most diverse superfamilies of cell surface receptors that sense a wide array of extracellular ligands, ranging from photons to small molecules and peptides [5]. Upon ligand binding, GPCRs activate the G-protein heterotrimer (Gαβγ) through the exchange of GDP for GTP on the Gα subunit, leading to the dissociation of Gα-GTP and Gβγ [6,7]. These G proteins then modulate downstream signalling by activating kinases, lipases, GTPases and ion channels, which govern cellular physiology [8]. The physiological outcomes of activated G proteins depend on the type of G-protein coupling of the receptors and the Gβγ composition of the cell. For instance, heterotrimers with Gαq, 12/13 signals through phospholipase C (PLC) and RhoA pathways [9,10], while Gαs and Gαi control the secondary messenger cAMP [11,12]. Although all heterotrimeric pathways produce Gβγ, the signalling intensity of Gβγ is more prominent upon activation of Gαi/o and Gαs heterotrimer [13]. Most cells show highly downregulated expression of Gαq, and thus the concentration of Gβγ generated upon activation of this pathway is relatively minor [14]. Though initially considered a passive signalling regulator of Gα activity, during the last two decades, Gβγ has been identified as a major signalling actuator that controls numerous effector pathways, including G-protein-gated inwardly rectifying K^+^ channels, adenylyl cyclase, PLC, phosphoinositide 3-kinase γ (PI3Kγ) and GPCR kinases [15–19].

Although GPCR-mediated activation of G-protein heterotrimers is considered the major active regulator of numerous cellular activities in response to external stimulation [1,20–23], it is understood that activities of the same downstream signalling components, albeit at lower intensities, are required for the ambient signalling of cells [24]. Recent studies have indicated intricate molecular processes beyond the canonical GPCR-based model consisting of alternative forms (accessory proteins) of heterotrimeric G-protein activity modulation independent of receptor activation [25,26]. Activators of G-protein signalling (AGS) family proteins are one such major regulators. Evidence indicates that AGS family members impose several distinct modes of G-protein heterotrimer regulation and invoke GPCR-independent G-protein signalling [27]. AGS family proteins consist of three different classes of proteins based on their mechanism of action: (i) class I AGS proteins mainly function as guanine nucleotide exchange factors (GEFs) to induce GDP to GTP exchange and heterotrimer activation (like GPCRs); (ii) class II AGS proteins function as guanine nucleotide dissociation inhibitors (GDIs) as their specific G-protein regulatory motifs (GPR) facilitate the binding to inactive GαiGDP form and repel Gβγ independent of GPCR activation; and (iii) class III AGS proteins tend to interact with heterotrimers and promote effector activation, which is independent of the nucleotide exchange [28–30]. As many G-protein functions are evaluated by adding extracellular ligands for their respective GPCRs, the inability to use such a method to trigger and detect the action of AGS family proteins has been an obstacle to a clear understanding of their signalling and regulation. To overcome this, we used protein engineering to generate a novel OptoGDI capitalizing on the light-sensitive cryptochrome 2 photolyase homology (CRY2PHR) domain in combination with the more efficient consensus GPR motif derived from AGS3 [29,31]. Using OptoGDI, we show for the first time the real-time activity of a GDI protein in living cells. We also show the feasibility of using this approach to optically invoke standalone subcellular Gβγ signalling, including PI3K activation and directional cell migration. The presented data indicate that this novel optogenetic approach will allow spatiotemporal control over various crucial Gβγ-mediated signalling processes, such as the regulation of ion channels and kinases independently of canonical GPCR signalling pathways [32–35] and therefore elucidate molecular underpinnings of complex regulatory networks governing cell signalling and behaviours. Such findings can potentially advance our understanding of fundamental cellular processes and offer new avenues for therapeutic intervention.

## 2. Material and methods

### 2.1. Reagents

Reagent sources: norepinephrine (Sigma-Aldrich, St. Louis, MO), Gallein (TCI AMERICA), Wortmannin and Bombesin (Cayman Chemical, Ann Arbor, MI), 11-*cis*-retinal (National Eye Institute, Bethesda, MD). All reagents except 11-*cis*-retinal (ethanol) were initially dissolved in DMSO and then diluted in HBSS or cell culture medium before adding to cells.

### 2.2. DNA constructs

DNA constructs: blue opsin [36] has been described previously [37]. α2AR-CFP, Venus-Gγ9, Split Venus β2γ9, Akt-PH-Venus, Akt-PH-mCh, Venus-PH, CRY2-mCh and CRY2-mCh-GPRcn constructs were kindly provided by the laboratory of Prof. N. Gautam at Washington University School of Medicine, St. Louis, MO. GRPR was a kind gift from the laboratory of Zhou-Feng Chen [38]. The parent construct of AGS3 was used to PCR out GPR-IV and inserted to the linearized CRY2-mCh at its C terminus using Gibson assembly followed by Dpn1 reaction. CRY2-mCh-AGS3-GPR-IV was PCR amplified using appropriate primers with overhangs containing expected nucleotide mutations to generate CRY2-mCh-GPRcn (1×) consensus. This construct was the parent to add subsequent multiple consensus sequences to generate CRY2-mCh-GPRcn (3×) and CRY2-mCh-GPRcn 6× (OptoGDI). To create Lyn-CIBN, we linearized a parent construct with Lyn at the N terminus and incorporated the PCR-amplified CIBN at the C terminus. All cloning was performed using Gibson assembly cloning (NEB). All cDNA constructs were confirmed by sequencing.

### 2.3. Cell culture and transfections

RAW264.7, HeLa and HEK293T cells were initially purchased from ATCC, USA. Recommended cell culture media: RAW264.7 (RPMI/10% DFBS/1% PS), HeLa (MEM/10% DFBS/1% PS) and HEK293T (DMEM/10% FBS/1% PS) were used to subculture cells at 70–80% (using versene-EDTA) on 29, 60 or 100 mm cell culture dishes in a humidified incubator at 37°C, 5% CO_2_. For live-cell imaging experiments, cells were seeded on 29 mm glass-bottomed dishes at a density of 1 × 10^5^ cells. DNA transfections were performed using either Lipofectamine 2000 reagent (for HeLa cells) or electroporation (for RAW264.7 cells) according to the manufacturer’s recommended protocols. Briefly, for electroporation of RAW264.7 cells, the following method was used. Nucleofector solution (82 µl), supplement solution (18 µl) from Amaxa Cell Line Nucleofector Kit V and appropriate volumes of plasmid DNA for specific DNA combinations were mixed. In each electroporation experiment, approximately 2 million cells were electroporated using the T-020 method of the Nucleofector 2b device (Lonza). Immediately after electroporation, cells were mixed with the cell culture medium at 37°C. Then, cells were centrifuged at 2800 r.p.m. for 3 min. Afterward, the cell pellet was re-suspended in appropriate volumes of cell culture media at 37°C and seeded on glass-bottomed dishes, 200 μl per well. After 2 h, 800 μl of more new media was added at 37°C. Imaging was conducted after approximately 5−6 h post-transfection, considering the high expression of constructs. HEK293T cells were plated in a six-well plate at the density of 2 million cells per well. After 2 h, cells were treated with 1 ml of complete media and transfected with respective DNA combinations (OptoGDI—1.5 μg, Gα-Rluc8—0.5 μg, Gβ—0.5 μg and GFP2-Gγ—0.5 μg) with 3.2 μl of PolyJet (SigmaGen) according to manufacturer’s instructions. Then, the six-well plate covered with aluminium foil was kept in a humidified incubator at 37°C, 5% CO_2_ overnight. The next day, transfected cells were washed with PBS once and detached using versine-EDTA (1 ml per well). Detached cells were centrifuged at 200*g* for 4 min and then re-suspended in complete DMEM media. Cells were then seeded at 0.1 million cells per well in 96-well plates coated with poly-D-lysine (100 μg ml^−1^). Plates were wrapped again with foil and kept in a humidified incubator overnight to minimize exposure of cells to light during this process.

### 2.4. Live-cell imaging, image analysis and data processing

The methods, protocols and parameters for live-cell imaging are adapted from previously published work [13,39,40]. Briefly, live-cell imaging experiments were performed using a spinning disc confocal imaging system (Andor Technology) with a 60×, 1.4 NA oil objective and iXon ULTRA 897BVback-illuminated deep-cooled EMCCD camera. Photoactivation and spatiotemporal light exposure on cells in regions of interest (ROI) was performed using a laser combiner with a 445 nm solid-state laser delivered using Andor FRAP-PA (fluorescence recovery after photobleaching and photoactivation) unit in real-time, controlled by Andor iQ 3.1 software (Andor Technologies, Belfast, United Kingdom). Fluorescent proteins such as CRY2-mCh, CRY2-mCh-AGS consensus 1×, CRY2-mCh-AGS consensus 3×, OptoGDI, Akt-PH-mCh were imaged using 594 nm excitation—624 nm emission settings. Akt-PH-Venus, Venus-PH and Split Venus β2γ9 were imaged using 515 nm excitation and 542 nm emission. α2AR-CFP was imaged using 445 nm excitation and 478 nm emission, respectively. For global and confined optical activation of CRY2-expressing cells, a 445 nm solid-state laser coupled to FRAP-PA was adjusted to deliver 145 nW power at the plane of cells, which scanned light illumination across the ROI at 1 ms μm^−2^. The time-lapse images were analysed using Andor iQ 3.2 software by acquiring the mean pixel fluorescence intensity changes of the entire cell or selected area/ROIs. Briefly, the background intensity of images was subtracted from the intensities of the ROIs assigned to the desired areas of cells (plasma membrane, endomembranes and cytosol) before intensity data collection from the time-lapse images. The intensity data from multiple cells were opened in Excel (Microsoft Office) and normalized to the baseline by dividing the whole dataset by the average initial stable baseline value. Data were processed further using Origin-Pro data analysis software (OriginLab).

### 2.5. BRET2 assay

After keeping cells in a 96-well plate overnight, plates were decanted and treated with 90 μl of BRET buffer (20 mM HEPES, 1× HBSS, pH 7.4) per well. Then, 10 μl coelenterazine 400a (Nanolight Technology) was added to each well at a final concentration of 2.5 ng μl^−1^. After 2  min incubation under dark, plates were read on the Synergy H1 plate reader (Biotek) with 410  nm (RLuc8-coelenterazine 400a) and 515 nm (GFP2) emission filters for 1  s integration time per well. After five measurements, plates were ejected, exposed to blue light for 30 s and continued for another 10 measurements. The GFP to Rluc8 ratio was calculated before and after blue light exposure, and the difference in BRET before and after blue light exposure was plotted using Origin-Pro data analysis software.

### 2.6. Determination of AGS3 consensus sequence structure and interactions with Gαi via AlphaFold and Schrodinger

**Figure 1.**
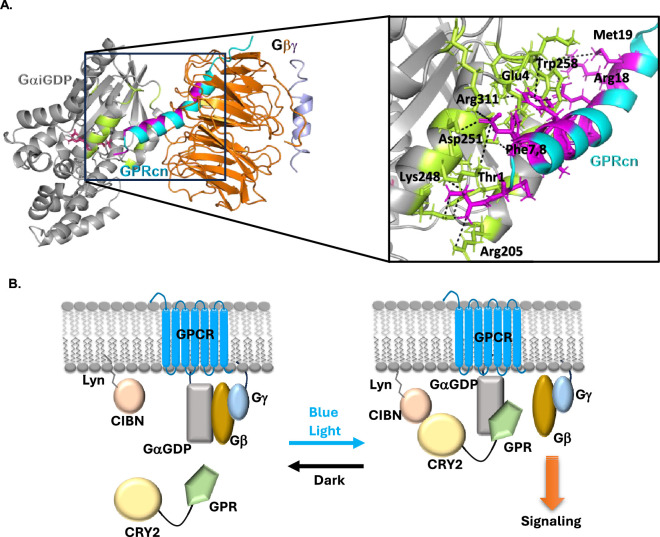
AGS3-GPR consensus peptide interacts with Gαi subunit. (A) Diagram depicting the interactions between GαiGDP–Gβγ and GαiGDP–GPR consensus peptide. GαiGDP—grey, Gβ—orange, Gγ—light blue, GPR motif—cyan. GαiGDP residues that interact with GPR peptide are shown in green. GPR residues that interact with GαiGDP are shown in pink (PDB ID 7E9H and 2V4Z). (B) Scheme for optical recruitment of cryptochrome 2-based GPR peptide to the plasma membrane.

We used the amino acid sequence of the AGS3 consensus sequence [41] to generate the model protein structure in figure 1A using AlphaFold2 [42]. The structure that best fits the currently available experimental information was selected for protein folding validation and optimization using the protein preparation tool and loop refinement tool in Schrodinger Bioluminate. Next, we incorporated a Gαi crystal structure available in the PDB database into Schrodinger; we cleaned the structure by removing the unwanted structures, ligands and solvents and prepared the protein using the protein preparation wizard. Next, we docked the prepared proteins using the Protein–Protein docking tool. The best hit with the lowest docking score was selected for further interpretations and to map interactions with Gαi.

### 2.7. Experimental rigour and statistical analysis

To eliminate potential biases or preconceived notions and improve the experimental rigour, we used the reagent-blinded-experimenter approach for the key findings of our study (i.e. Venus Gγ9 and Split Venus β2γ9 translocation assays). Additionally, OptoGDI-induced cell migration was conducted by two different experimenters. All experiments were repeated multiple times to test the reproducibility of the results. Results are analysed from multiple cells and represented as mean ± s.d. The respective figure legends give the exact number of cells used in the analysis. Digital image analysis was performed using Andor iQ 3.1 software, and fluorescence intensity obtained from ROI was normalized to initial values (baseline). Data plot generation and statistical analysis were done using OriginPro software (OriginLab). One-way ANOVA statistical tests were performed using OriginPro to determine the statistical significance between two or more populations of signalling responses. Tukey’s mean comparison test was performed at the *p* < 0.05 significance level for the one-way ANOVA statistical test. After obtaining the normalized data, PIP2 recovery rates were calculated using the NonLinear Curve Fitting tool in OriginPro. In the NonLinear Curve Fitting tool, each plot was fitted to the DoseResp (dose-response) function under the pharmacology category by selecting the relevant range of data to be fitted. The mean values of hill slopes (*P*) obtained for each curve fitting are presented as the mean rates of PIP2 recovery.

## 3. Results

### 3.1. Engineering of CRY2-based OptoGDIs

Our goals were to examine the regulatory role of AGS3 in signal processing in living cells and to create an optogenetic strategy to control standalone Gβγ signalling. The approach aimed to present an AGS3-derived Gα binding domain on optical command to the heterotrimer at the plasma membrane, releasing Gβγ. AGS3 is identified as a GDI and possesses four GPR motifs, which mediate its binding to the inactive Gαi/oGDP in the heterotrimer [29]. The need for a significant excess Gβγ to disrupt the Gα–AGS3 complex indicated that AGS3 could release Gβγ without GDP to GTP exchange at Gα [43].

Four GPR motifs in AGS3 may enhance AGS3-heterotrimer reaction cross section and thus provide effective separation of Gβγ from GαGDP. A consensus GPR motif (GPRcn) was proposed by aligning the four GPR repeats from five species [41]. GPRcn possesses an N-terminal negative charge from a *Glu* duo, followed by a hydrophobic cluster composed of *Phe* duo, *Leu* duo and several C-terminal hydrophilic residues (*Asp–Asp–Gln–Arg*) and has been tenfold effective in promoting heterotrimer dissociation than the Gβγ hotspot binding peptide, SIGK [44].

**Figure 2.**
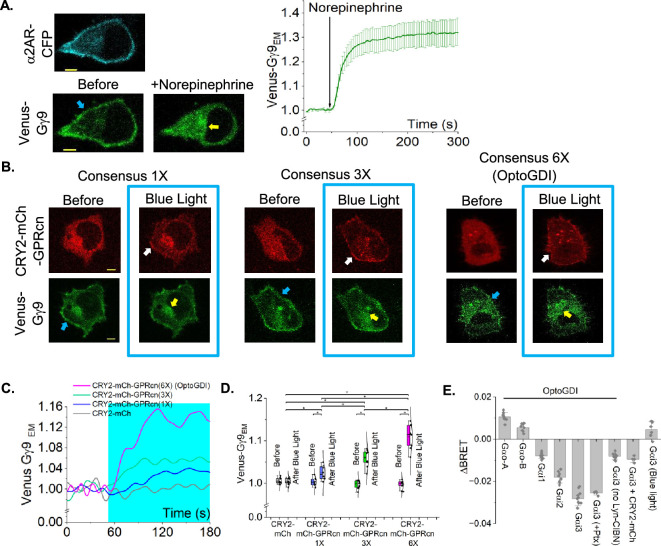
OptoGDI induces Gβγ translocation upon blue light exposure. (A) HeLa cells expressing α2AR-CFP and Venus-Gγ9 show robust Gβγ translocation upon 100 mM norepinephrine addition. The plot shows the baseline normalized Venus fluorescence in endomembranes over time. Yellow arrows indicate the accumulation of Gγ9 in internal membranes. The Venus-Gγ9 loss from the plasma membrane is indicated by the blue arrow (*n* = 10). (B) HeLa cells expressing Venus-Gγ9, Lyn-CIBN and CRY2-mCh-GPRcn (1×), CRY2- mCh-GPRcn (3×) or CRY2-mCh-GPRcn (6×; OptoGDI) show different extents of Venus-Gγ9 translocation to the endomembranes, upon blue light exposure. Yellow arrows indicate the accumulation of Gγ9 in internal membranes. The white arrow indicates the plasma membrane recruitment of CRY2-mCherry-GPRcn variants. The blue arrow indicates the Venus-Gγ9 loss from the plasma membrane. (C) The plot shows the baseline normalized Venus fluorescence in endomembranes over time (*n* = 15 for 1×, *n* = 14 for 3×, *n* = 17 for Opto-GDI). (D) The whisker box plot compares Gγ9 translocation extents to the basal Gγ9 fluorescence at endomembranes. Blue box indicates the blue light exposure. Average curves were plotted using cells from ≥3 independent experiments. (E) The bar chart shows the ΔBRET between Rluc8-tagged Gα and GFP2-tagged γ9 under different experimental conditions. OptoGDI with Gα0-A (*n* = 8), OptoGDI with Gα0-B (*n* = 8), OptoGDI with Gαi1 (*n* = 8), OptoGDI with Gαi2 (*n* = 8), OptoGDI with Gαi3 (*n* = 8), OptoGDI with Gαi3 and Ptx (*n* = 4), OptoGDI with Gαi3 but no Lyn-CIBN (*n* = 8), OptoGDI with CRY2-mCh (*n* = 4) and Gαi3 with blue light (*n* = 8). The error bars represent s.d. (standard deviation). The scale bar = 5 µm. CFP: cyan fluorescent protein; mCh: mCherry; EM: endomembranes; Ptx: Pertussis toxin.

Consistent with the current predictions based on the primary sequence analysis, using *in silico* modelling, we show that the consensus sequence derived from the 4^th^ GPR motif of AGS3 is likely to adopt an alpha-helical conformation [41,45]. Furthermore, our protein–protein docking data show a tight binding affinity between the modelled consensus GPR motif and GαiGDP, indicated by the several key interactions. Out of 30 potential configurations, more than 40% of configurations showed that the GPR helix binds to the same Gαi region. In particular, we found interactions between Thr1, Met2, Glu4, Phe7, Phe8, Leu10, Leu11, Ser14, Gln15, Arg18 and Met19 of the GPR with Arg205, Arg208, Ile212, Phe215, Glu216, Glu245, Lys248, Leu249, Asp251, Ser252, Asn256, Lys257, Trp258, Phe259 and Arg311 of GαiGDP, respectively. Based on our modelled structure, the GPR motif is likely to be an amphipathic helix (figure 1A). Using AGS3 full sequence and site-directed mutagenesis, we generated GPRcn and fused it to the C terminus of *Arabidopsis* CRY2-PHR tethered mCherry [31]. Similar to the full wild-type AGS3 protein, CRY2-mCherry-GPRcn also showed a cytosolic distribution. Upon blue light exposure, CRY2PHR binds to CIBN [31]. Since the goal was to generate Gβγ at the plasma membrane on an optical command, we generated N-terminally myristoylated/palmitoylated CIBN (Lyn-CIBN) using CIBN-CAAX (figure 1B). Upon blue light illumination, CRY2-mCherry-GPRcn was recruited to the plasma membrane, indicating its heterodimerization with CIBN (figure 1B, right). To determine whether GPRcn interacts with plasma membrane-bound Gαi/oGDP and repel Gβγ, we employed the Gβγ9 translocation assay [13,46]. We have previously established GPCR activation-induced Gγ9 translocation (as Gβγ complex) from the plasma membrane to endomembranes as an assay to measure GPCR–G-protein activity in living cells [46]. As a positive control, we show the robust Gγ9 translocation exhibited by HeLa cells expressing Venus-Gγ9 upon activation of α2AR-CFP with 100 μM norepinephrine (figure 2A).

We first examined the blue light-induced activity of CRY2-mCherry-GPRcn (1×), which only contains one GPRcn, in HeLa cells also expressing Lyn-CIBN and Venus-Gγ9. Upon blue light-induced plasma membrane recruitment of CRY2-mCherry-GPRcn (1×; figure 2B, white arrow), cells showed a minor yet significant Gγ9 translocation (figure 2B, left, plot-blue curve; one-way ANOVA, *F*_1, 18_ = 60.49, *p* ≤ 0.0001, electronic supplementary material, table S1A,B) unlike GPCR activation-induced translocation (figure 2A). We believe an inefficient binding affinity for GαGDP and lack of proper spatial orientation for the interaction to be few of the several possible reasons underlying the optogenetic GDI-induced minor Gγ9 translocation. Since AGS3 consists of four GPR motifs at its C terminus, we hypothesized that increasing the number of consensus repeats in the optogenetic module may trigger a higher extent of Gβγ release from the heterotrimer. Therefore, we generated two new constructs, CRY2-mCherry-GPRcn (3×) and CRY2-mCherry-GPRcn (6×), in which we inserted flexible linker sequences between the consensus peptides. Upon blue light exposure, CRY2-mCherry-GPRcn (3×) liberated a detectable amount of free Gβγ, while CRY2-mCherry-GPRcn (6×) exhibited significantly higher free Gβγ generation, measured using the change in endomembrane fluorescence due to Venus-Gγ9 translocation (figure 2B, middle and right images, plot-green and pink curves). Additionally, we performed a similar experiment using CRY2PHR-mCherry, which does not contain a GPRcn motif at its C terminus to confirm that the observed Venus-Gγ9 is due to the Gβγ liberation from the GPRcn recruitment. Upon blue light-induced plasma membrane recruitment of CRY2-mCherry (electronic supplementary material, figure S1A, top), cells did not show a detectable Gγ9 translocation (electronic supplementary material, figure S1A, bottom and figure 2C,D, grey line and box). One-way ANOVA showed that CRY2-mCherry- GPRcn (6×) induces significantly higher Gβγ liberation than that of the other two versions (figure 2D, whisker box plot; one-way ANOVA, *F*_2, 24_ = 25.04, *p* ≤ 0.0001, electronic supplementary material, table S4A,B). Therefore, the studies henceforward are performed using CRY2-mCherry-GPRcn (6×), which we named OptoGDI. To investigate the Gαi/o subtype specificity of OptoGDI towards different heterotrimers, we used TRUPATH biosensors in which the heterotrimer dissociation can be detected using bioluminescence energy transfer between Rluc8 incorporated specific Gα type and GFP2-tagged Gγ9 [47]. OptoGDI effectively generated free Gβγ from Gαi2 and Gαi3 heterotrimers upon exposure to blue light (figure 2E). However, OptoGDI could not liberate Gβγ from Gαi1, Gαo-A and Gαo-B heterotrimers. The effect of OptoGDI on Gαi3 heterotrimer dissociation was not disturbed in cells treated overnight with pertussis toxin (200 ng ml^−1^), indicating such Gβγ generation is nucleotide exchange and Gαi-coupled GPCR independent. Control experiments with only OptoGDI showed minimum activity without its binding partner, Lyn-CIBN. Similarly, another control experiment with CRY2-mCh with Lyn-CIBN confirmed that the GDI portion is required for Gβγ liberation from Gαi-3 heterotrimers. Additionally, we did not observe any BRET signal after blue light stimulation of cells only expressing Gαi-3 heterotrimers without OptoGDI, confirming OptoGDI is required to liberate Gβγ.

### 3.2. OptoGDI-induced subcellular free Gβγ generation



**Figure 3.**
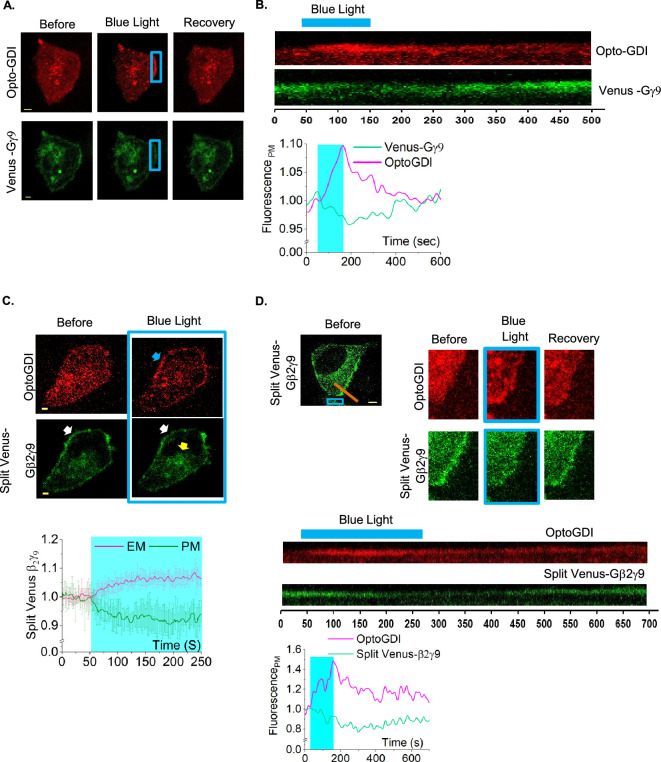
OptoGDI induces subcellular free Gβγ generation upon blue light exposure. (A) Confined membrane region of a HeLa cell expressing OptoGDI, Lyn-CIBN and Venus-Gγ9 was exposed to blue light. The cell exhibited an OptoGDI recruitment and Gγ9 translocation only on the blue light-exposed region of the cell (*n* = 12). (B) The kymographs and the plot showing the blue light-induced OptoGDI recruitment and the Gγ9 loss and the recovery on the plasma membrane. (C) HeLa cells expressing Opto-GDI, Lyn-CIBN and Split Venus-Gβ2γ9 show detectable free Gβγ translocation upon blue light exposure. The blue arrow at the membrane indicates the OptoGDI recruitment after blue light stimulation. The white arrow indicates Split Venus-Gβ2γ9 fluorescence loss from the plasma membrane. The plot shows the baseline normalized Venus fluorescence in endomembranes and the plasma membrane over time (*n* = 8). (D) HeLa cells expressing OptoGDI, Lyn-CIBN and Split Venus-Gβ2γ9 show subcellular free Gβγ generation upon blue light exposure to a confined membrane region. A kymographic view of the respective region of the cell shows OptoGDI and Split Venus-Gβ2γ9 dynamics at the plasma membrane over time. The orange line on the cell image indicates the membrane region used to create the kymograph. (*n* = 12). Yellow arrows indicate the Venus fluorescence increase on the plasma membrane when Opto-GDI is cytosolic. Average curves were plotted using cells from≥3 independent experiments. The blue box indicates blue light exposure. The error bars represent s.d. (standard deviation of mean). The scale bar = 5 µm.

Previous studies have shown the utility of several optogenetic tools to spatiotemporally control G-protein signalling in subcellular locations upon light stimulations [36,48–50]. Therefore, next, to examine OptoGDI-induced subcellular G-protein signalling, we examined the ability of OptoGDI to induce subcellular Gβγ release and subsequent signalling. In HeLa cells expressing Lyn-CIBN, OptoGDI and Venus-Gγ9, OptoGDI showed cytosolic, and Venus-Gγ9 displayed primarily plasma membrane distribution (figure 3A, left). We then exposed a selected sub-plasma membrane region to blue light using a localized blue light stimulus (445 nm, 26.3 μW µm^−2^; figure 3A, middle—blue box). Robust recruitment of OptoGDI to the blue light-exposed region of the cell and subsequent localized loss of Venus-Gγ9 membrane fluorescence, indicating Gβγ translocation (figure 3A, middle). Upon termination of the blue light, OptoGDI dissociation from the plasma membrane and Gγ9 recovery was observed, suggesting that OptoGDI induced a reversible heterotrimer dissociation (figure 3A, right, B-Kymographs and the plot). Additionally, the kymograph shows OptoGDI and Venus-Gγ9 localizations at the plasma membrane are inversely synchronized (figure 3B, kymographs and plot).

To ascertain that the observed Gγ9 translocation upon OptoGDI recruitment to the plasma membrane is not an experimental artefact and truly due to Gβγ liberation from the heterotrimer, we used a fluorescent complementation approach to track the distribution of both Gβ and Gγ. We expressed OptoGDI, Lyn-CIBN, Venus (1–155)-γ9 and Venus (155–209)-β2 in HeLa cells [51]. It has been shown that the two proteins must be proximal enough to assemble the fluorescence protein Venus [52]. Venus (1–155)-γ9 and Venus (156–209)-β2 do not exhibit any fluorescence when they are not interacting [53]. HeLa cells expressing the above constructs combination showed a Venus fluorescence with a reasonable plasma membrane localization (figure 3C, bottom), while OptoGDI was observed in the cytosol. Next, we recruited OptoGDI to the plasma membrane by exposing cells to blue light (figure 3C, top images—blue arrow). Venus fluorescence on the plasma membrane was reduced (figure 3C, white arrow) with a corresponding fluorescence increase in endomembranes (figure 3C, yellow arrow and the plot). In the Gβγ dimer, the N-termini of Gβ and Gγ are only a few angstroms apart, allowing for Venus fluorescence appearance due to complementation. Since Gβγ is a stable dimer, the Venus fluorescence remained intact upon OptoGDI-induced Gβγ translocation as well. Next, we examined the feasibility of liberating subcellular free Gβγ by using Split Venus Gβγ. We exposed a confined region of the plasma membrane to blue light and recruited OptoGDI (figure 3D). We observed the loss of Venus fluorescence at the blue light-exposed plasma membrane region, confirming Gβγ liberation from the heterotrimer. Upon blue light termination, OptoGDI gradually returned to the cytosol. A synchronized reverse translocation of Gβγ to the plasma membrane was observed, as indicated by Venus fluorescence recovery. The images and kymograph clearly indicate that the observed forward and reverse translocation of Gβγ was determined by the presence and the absence of OptoGDI at the plasma membrane, respectively (figure 3D, cell images and kymograph). This data also indicated that OptoGDI-induced Gβγ release is reversible.

### 3.3. OptoGDI-liberated Gβγ modulates PLCβ-induced PIP2 hydrolysis

**Figure 4.**
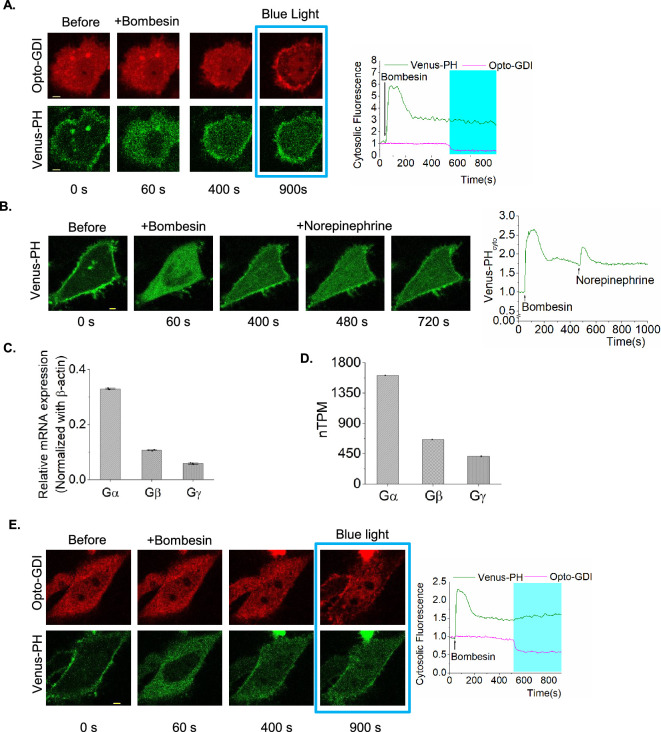
OptoGDI induces secondary PIP2 hydrolysis in Gq-GPCR-activated cells. (A) HeLa cells expressing GRPR, Venus-PH, OptoGDI and Lyn-CIBN exhibited robust PIP2 hydrolysis upon 1 μM bombesin addition. OptoGDI was then recruited to the plasma membrane after the PIP2 hydrolysis reached equilibrium. The cells did not exhibit a rescue of PIP2 hydrolysis (*n* = 15). (B) GRPR in HeLa cells expressing α2AR-CFP and Venus-PH was activated using 1 μM bombesin, and the cells exhibited robust PIP2 hydrolysis. α2AR was then activated using 100 μM norepinephrine after the PIP2 hydrolysis reached the equilibrium. The cells exhibited robust secondary PIP2 hydrolysis, indicating the PIP2 hydrolysis rescue (*n* = 10). (C) Normalized relative mRNA expression level from RNA-seq data of HeLa cells showing elevated expression of endogenous G-protein α compared to G-protein β and γ, normalized to the mRNA expression level of β-actin. (*n* = 3). (D) Normalized transcripts per million (nTPM) levels of human G-protein α, β and γ levels from Human Protein Atlas showing the elevated level of G-protein α, compared to the G-protein β and γ. (E) HeLa cells expressing GRPR, Venus-PH, Gβ1, Lyn-CIBN and OptoGDI were exposed to 1 μM bombesin. OptoGDI was then recruited to the plasma membrane of the cell after the PIP2 hydrolysis reached equilibrium. The cell showed detectable secondary PIP2 hydrolysis as indicated by the Venus-PH translocation to the cytosol (*n* = 12). The scale bar = 5 µm. Blue box indicates the blue light exposure. GRPR: gastrin-releasing peptide receptor; PIP2: phosphatidylinositol 4,5-bisphosphate; PM: plasma membrane; PH: Pleckstrin homology; Cyto: cytosolic fluorescence.

Gq-coupled GPCR activation induces a self-attenuating PIP2 hydrolysis [10]. We and others showed that the dual stimulation from GαqGTP and Gβγ forms the highly effective lipase complex, GαqGTP–PLCβ–Gβγ, and the observed PIP2 hydrolysis attenuation is due to the loss of Gβγ from this complex through the activation of PLCβ and generation of the less effective lipase GαqGTP–PLCβ [10,54]. We also showed that PIP2 hydrolysis can be rescued by providing Gβγ to a system with PIP2 hydrolysis partially attenuated due to the loss of Gβγ from GαqGTP–PLCβ–Gβγ [10]. Here, we first examined whether Gβγ generated by membrane-recruited OptoGDI retards PIP2 hydrolysis attenuation in HeLa cells expressing GRPR (a Gq-coupled GPCR), Venus-PH (PIP2 sensor), Lyn-CIBN and OptoGDI upon activation of GRPR using 1 µM bombesin. After PIP2 hydrolysis reached equilibrium, we exposed cells to blue light and recruited OptoGDI to the plasma membrane. However, cells did not exhibit the expected PIP2 hydrolysis rescue that occurs due to free Gβγ generation (figure 4A). To confirm that Gβγ, freed from Gi/o GPCR activation, can rescue the diminished PIP2 hydrolysis under the same experimental conditions, we investigated the PIP2 hydrolysis recovery in HeLa cells expressing GRPR, α2AR-CFP and Venus-PH. The cells demonstrated characteristic PIP2 hydrolysis upon GRPR activation using 1 μM bombesin. After PIP2 recovery reached a steady state, the activation of α2AR with 100 µM norepinephrine induced significant re-hydrolysis of PIP2 (figure 4B), indicating that α2AR activation can facilitate the rescue of PIP2 hydrolysis.

Compared to Gβγ dimers released upon Gi/o-coupled GPCR activation (figure 2A), OptoGDI liberated only a fraction (figure 2B). To confirm that the limited free Gβγ generation is the underlying reason behind the lack of OptoGDI-induced PIP2 hydrolysis rescue observed (figure 4A), compared to the Gi/o-coupled GPCR-induced (figure 4B), we explored avenues to increase the concentration of liberated Gβγ by OptoGDI. In our analysis of endogenous G-protein expression levels in HeLa cells using RNA-seq data, we found that Gα exhibited a higher expression compared to Gβγ (figure 4C). Furthermore, data from the Human Protein Atlas confirmed this finding, displaying a similar trend (figure 4D). We hypothesized that some of the liberated Gβγ by OptoGDI is sequestered by the excess GαGDP on the plasma membrane. Our previous computation modelling also suggested a similar regulatory mechanism [13]. Therefore, to reduce free GαGDP availability, we additionally expressed Gβ in HeLa cells, also expressing GRPR, Venus-PH, OptoGDI and Lyn-CIBN. Upon GRPR activation, these cells showed robust PIP2 hydrolysis, which self-attenuated, reaching a steady state (figure 4E). Upon blue light-induced OptoGDI recruitment to the plasma membrane, a detectable rescue of PIP2 hydrolysis was observed, indicating that Gβγ freed at the plasma membrane by OptoGDI can enhance the lipase activity of PLCβ (figure 4E). In a similar control experiment, where we expressed CRY2-mCherry in the place of OptoGDI, blue light exposure failed to rescue PIP2 hydrolysis (electronic supplementary material, figure S2A).

**Figure 5.**
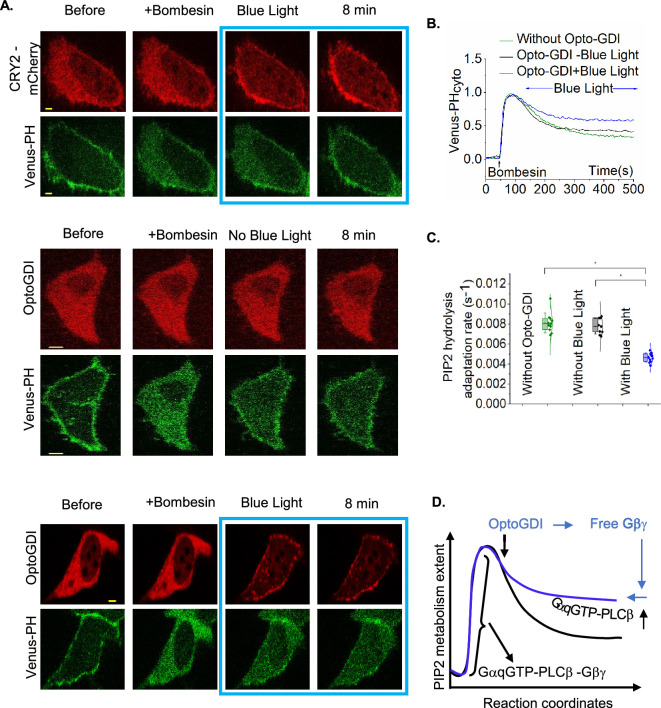
OptoGDI modulates PLCβ-induced PIP2 hydrolysis. (A) Top—GRPR, Venus-PH, Lyn-CIBN and CRY2-mCh were expressed in HeLa cells and cells were exposed to bombesin first and then exposed to blue light after the PIP2 hydrolysis reached the maximum. CRY2-mCh membrane-recruited cells exhibited the typical PIP2 hydrolysis response followed by a PIP2 hydrolysis attenuation (*n* = 11). Middle—HeLa cells expressing GRPR, Venus-PH, Lyn-CIBN and OptoGDI showed robust PIP2 hydrolysis upon bombesin addition. The PIP2 hydrolysis attenuation rates were also similar to the previous condition (*n* = 9). Bottom—HeLa cells expressing GRPR, Venus-PH, Lyn-CIBN and OptoGDI were first exposed to bombesin and then exposed to blue light, and OptoGDI was recruited to the plasma membrane when the PIP2 hydrolysis reached the maximum. The cells showed a robust PIP2 hydrolysis upon bombesin addition. The PIP2 hydrolysis attenuation rates were significantly lower than the control cells (*n* = 11). (B) The corresponding plot shows the PIP2 sensor dynamics in the cytosol of the cells. (C) The whisker box plot shows the statistical differences in PIP2 hydrolysis attenuation rates between the control cells and OptoGDI recruited cells. (D) The schematic shows the mechanism of PIP2 hydrolysis attenuation. The scale bar = 5 µm. Blue box indicates the blue light exposure. GRPR: gastrin-releasing peptide receptor; mCh: mCherry fluorescent protein; PIP2: phosphatidylinositol 4,5-bisphosphate; Cyto: cytosolic fluorescence; PH: Pleckstrin homology.

We next examined whether Gβγ generated by OptoGDI could reduce the rate of PIP2 hydrolysis attenuation that occurred after initial Gq-GPCR activation. Similar to the previous experiment, we examined GRPR-induced PIP2 hydrolysis in HeLa cells expressing GRPR, Venus-PH, Lyn-CIBN and either CRY2-mCherry (control) or OptoGDI upon activation of GRPR using 1 µM bombesin. Here, we employed two controls. In the first control, we examined PIP2 dynamics in cells where we recruited CRY2-mCherry to the plasma membrane using blue light exposure after PIP2 hydrolysis reached the maximum (figure 5A, top panel images and 5B plot—green curve). We then examined PIP2 dynamics without OptoGDI recruitment to the plasma membrane in the second control. Interestingly, both the controls showed similar PIP2 dynamics (figure 5A, middle, 5B and C). Blue light-induced OptoGDI recruitment to the plasma membrane after the maximum PIP2 hydrolysis significantly retarded the PIP2 hydrolysis attenuation compared to the control cells (figure 5A, bottom panel and 5B*,* blue curve). The whisker box plot shows that the rate of PIP2 hydrolysis attenuation is significantly reduced in cells with membrane-recruited OptoGDI (figure 5C; one-way ANOVA, *F*_2, 28_ =66.86, *p* = 2.188 × 10^−11^, electronic supplementary material, table S5A*,*B). Here, we believe that OptoGDI recruitment to the plasma membrane of GRPR-activated cells provides additional Gβγto retard the PIP2 hydrolysis attenuation due to the reaction GαqGTP–PLCβ–Gβγ ⇌ GαqGTP–PLCβ + Gβγ, in which Gβγ is lost due to translocation [10]. Therefore, we propose that OptoGDI pushes this reaction left, creating a more efficient lipase and reducing the PIP2 hydrolysis attenuation (figure 5D).

### 3.4. Localized OptoGDI induces subcellular PIP3 generation and macrophage migration

Asymmetric activation of Gi/o GPCRs due to a chemokine gradient induces directional cell migration [55,56]. We have shown that Gβγ signalling at the leading edge, primarily Gβγ-induced PI3Kγ activation, generates traction forces through PIP3-mediated cytoskeleton remodelling and actively triggers the retraction of the trailing edge, facilitating the relocation of the cell body towards the chemokine gradient or the asymmetric optical stimulation [48,57,58]. Here, we examined whether asymmetric GDI activity across a cell could trigger similar cell migration.

**Figure 6.**
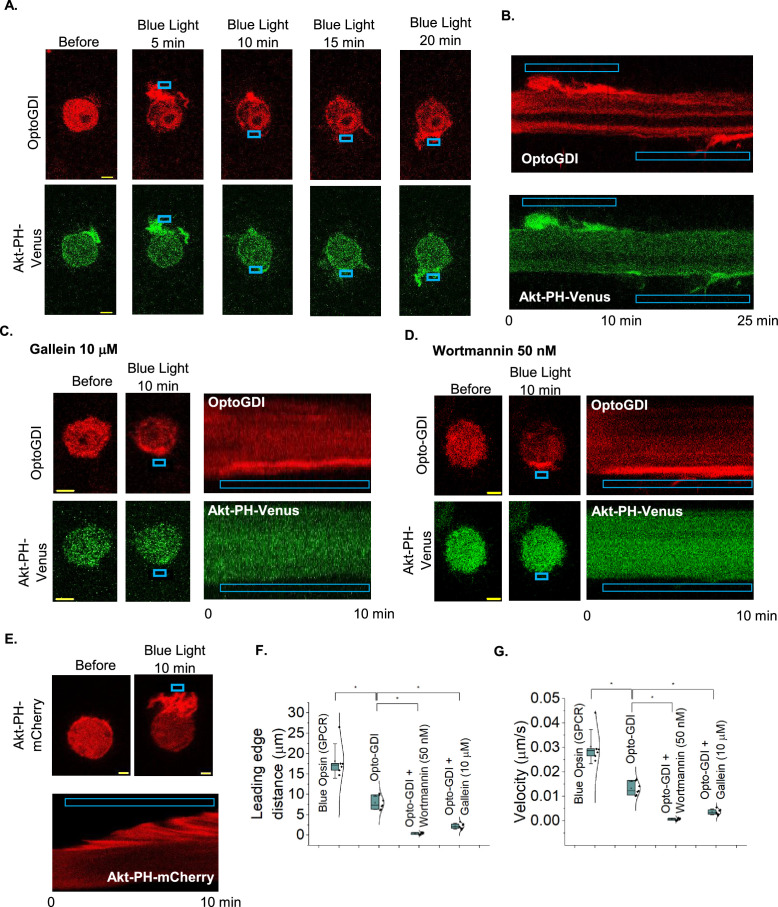
OptoGDI-triggered subcellular Gβγ induced PIP3 generation and macrophage migration. (A) RAW 264.7 cells expressing OptoGDI, Lyn-CIBN and AKT-PH-Venus were optically activated to recruit OptoGDI to a confined region (top). The cell shows localized PIP3 production (bottom images) and the optogenetic activation of PIP3 production resulted in a detectable cell migration response towards the blue light. The blue box indicates the photoactivation (*n* = 10). (B) Kymographs of the same cell show the accumulation of OptoGDI (red) and Akt-PH-Venus (green) in the leading edge of the migrating cell. (C) RAW 264.7 cells transfected with OptoGDI, Lyn-CIBN and Akt-PH-Venus were subjected to localized optical activation in the presence of 10 μM gallein (Gβγ inhibitor). Cells were incubated with the inhibitor for 30 min at 37°C before imaging and optical activation. The cells did not show a detectable migration or PIP3 generation. Kymographs of the same cell show the accumulation of OptoGDI (red) in the leading edge of a cell but no Akt-PH-Venus (green) accumulation (*n* = 10). The blue box indicates photoactivation. (D) RAW 264.7 cells transfected with OptoGDI, Lyn-CIBN and Akt-PH-Venus were subjected to localized optical activation in the presence of 50 nM wortmannin (PI3K inhibitor). Cells were incubated with the inhibitor for 30 min at 37°C before imaging and optical activation. The cells did not show a detectable migration or PIP3 generation. Kymographs of the same cell show the accumulation of OptoGDI (red) in the leading edge of the cell but no detectable accumulation of the Akt-PH-Venus (green). The blue box indicates the photoactivation (*n* = 12). (E) RAW 264.7 cells transfected with blue opsin and AKT-PH-mCherry were subjected to localized optical activation with confined blue light (blue box), after the 50 mM 11-*cis*-retinal treatment. The cell showed an optogenetic activation-induced localized PIP3 production and robust migration response towards the blue light. The kymograph of the same cell shows the accumulation of Akt-PH-mCherry (red) at the leading edge of the cell, indicating the PIP3 generation (*n* = 9). (F) The whisker plot shows the extent of movement of the peripheries of the leading edge of control and pharmacologically perturbed RAW 264.7 cells in C,D. (G) The whisker plot shows the extent of migration velocities of the peripheries of the leading edge of the RAW 264.7 cells in A*–*E. Average curves were plotted using cells from ≥3 independent experiments. The error bars represent s.d. (standard deviation of the mean). The scale bar = 5 µm.

We expressed OptoGDI, Akt-PH-Venus (PIP3 sensor) and Lyn-CIBN in RAW264.7 cells and then exposed a confined region of the cells to blue light (figure 6A top, blue box; electronic supplementary material, video S1), and cells not only showed OptoGDI recruitment to the blue light-exposed side of the cells but also an accompanying localized PIP3 generation (figure 6A, bottom; electronic supplementary material, video S2). The kymographic views of the cells clearly show the blue light-induced plasma membrane-localized OptoGDI, PIP3 generation, and the subsequent movement of the cell towards blue light (figure 6B). We used CRY2-mCherry as a negative control to confirm that our observations were due to membrane recruitment of GPRcn motifs, however, not because of CRY2 recruitment to the plasma membrane. We expressed CRY2-mCherry, Akt-PH-Venus and Lyn-CIBN in RAW264.7 cells and examined the PIP3 generation and cell behaviour using blue light-induced localized CRY2-mCherry recruitment. As expected, localized CRY2-mCherry recruitment to the plasma membrane (electronic supplementary material, figure S3A*,* top—blue box, Kymograph—top) did not show any detectable PIP3 generation or cell migration (electronic supplementary material, figure S3A*,* bottom—blue box, Kymograph—bottom).

As control experiments to ensure that the observed PIP3 generation is Gβγ mediated and cell migration is PI3K activation-dependent, we performed a similar experiment, however, either with cells exposed to 10 µM gallein, a Gβγ inhibitor [48], or 50 nM wortmannin, a PI3K inhibitor [48]. In both control experiments, although the localized blue light-induced subcellular OptoGDI recruitment, cells neither exhibited PIP3 generation nor directional cell migration (figure 6C,D). The OptoGDI-induced RAW264.7 cell migration extent and velocities observed in gallein, and wortmannin-treated cells were also significantly lower than in the untreated control cells (figure 5E,F; one-way ANOVA, *F*_1, 11_=120.35, *p* ≤ 0.0001, electronic supplementary material, table S10A,B; and one-way ANOVA, *F*_1, 12_ = 71.59, *p* ≤ 0.0001, electronic supplementary material, table S11A,B). As a positive control, we next compared the observed OptoGDI-induced RAW264.7 cell migration with blue opsin, a Gi/o-coupled GPCR, activation-induced macrophage migration. We expressed blue-opsin and Akt-PH-mCherry in RAW264.7 cells, which are exposed to 500 nM 11-*cis*-retinal before exposing cells to localized blue light activate opsins in one side of the cell. The cells showed robust migration towards blue light while forming lamellipodia at the activated leading edge of the cell (figure 6C, cell images and kymograph). Upon blue opsin activation, Akt-PH-mCherry accumulation at the leading edge of the cell further indicated the robust PIP3 generation. One-way ANOVA indicated that the OptoGDI-induced RAW264.7 cells migration (distances and the migration velocities) is significantly lower than that of blue opsin-induced (figure 6E,F; one-way ANOVA, *F*_1, 11_ =35.35, *p* ≤ 0.0001, electronic supplementary material, table S6A,B; and one-way ANOVA, *F*_1, 11_ =35.35, *p* ≤ 0.0001, electronic supplementary material, table S9A,B). These data collectively indicate that, though less potent than GPCR-induced, OptoGDI-triggered subcellular Gβγ can also induce localized PIP3 generation significant enough to cause directional macrophage migration independent of GPCR activation.

## 4. Discussion

In this study, we demonstrate *in silico* structure-guided engineering of optogenetic GDI utilizing the AGS3 consensus GPR motif, enabling precise optical control of GDI-heterotrimer interactions to release signalling active Gβγ. Building on our previous work highlighting the substantial involvement of free Gβγ in PIP2 hydrolysis and its attenuation [10], we here demonstrated a negative impact on the canonical PIP2 hydrolysis attenuation observed upon Gq-GPCR activation through the OptoGDI-induced optogenetic release of free Gβγ. Using a Gγ subunit that provides the Gβγ complex the lowest observed membrane affinity, Gγ9 [59,60], we show the reversible Gβγ release in single cells and from subcellular regions. To our understanding, this is the first direct demonstration of real-time GDI signalling in living cells. Furthermore, we show that spatially and temporally precise optical recruitment of OptoGDI to subcellular regions of the plasma membrane effectively initiates Gβγ-mediated signalling events, including localized PIP3 generation and associated macrophage migration. Although there are many studies on Gi/o GPCR activation-induced and primarily Gβγ-mediated cell migration, the work presented here shows that asymmetric standalone Gβγ signalling is sufficient to induce effective directional cell migration. As anticipated from a constitutively active signalling regulator, though the OptoGDI-induced Gβγ generation and subsequent signalling were relatively weaker than GPCR-induced, the resultant signalling remained significant. The reversible switching of Gbg signalling using OptoGDI was shown through the reversible translocation of Gbg, PIP3 generation and directional cell migration by optically controlling the activity of OptoGDI at the plasma membrane.

The influence of AGS3 on a variety of signalling processes has been demonstrated. Among them, AGS3-influenced protein trafficking to the plasma membranes [61], μ-opioid receptor-induced PKA signalling in primary striatal neurons [62] and the phosphorylation of cyclic AMP response element-binding protein associated anti-apoptotic effects [63] are significant. The ability of the GPR motif of AGS3 to bind the G-protein heterotrimers, specifically GαGDP, through the upstream negatively charged and hydrophobic residues of the GPR motif liberating Gβγ has been suggested as the primary underlying molecular reasoning for AGS3 signalling [41]. Also, it has been observed that the Gαi-coupled GPCRs do not efficiently couple to GPR-bound GαiGDP, suggesting that although the receptor interacts with the GαiGDP–GPR complex, this interaction stabilizes a receptor conformation that has a low affinity for agonist [41]. It is also unclear whether GPR motif binding to GαGDP of the heterotrimer always results in free Gβγ release or whether GPCRs can activate GPR-bound heterotrimers. Furthermore, whether AGS3-heterotrimer interaction is a negative regulator for GPCR–G-protein signalling and whether the GαiGDP–GPR complex acts as an active signalling entity are also unclear. Not only does the present study shed some light on these questions, but it has also provided a valuable avenue to understand AGS3 signalling in particular and GDI signalling in general. Here, we also introduce an optogenetic tool that is compatible with subcellular applications, allowing for precise spatial and temporal control of standalone Gβγ signalling.

Collectively, our *in silico* structural analysis indicates the GPR motif binding the GαGDP–Gβγ interface, while the provided single cell and subcellular experimental data show that OptoGDI induces the release of signalling active Gβγ, providing a molecular picture and mechanistic basis underlying AGS3 signalling. We show the unique capabilities of a novel optogenetic GDI for spatially and temporally precise release of Gβγ in specific subcellular plasma membrane regions of living cells, thereby initiating a localized key signalling event in PIP3 generation and a crucial physiological response leading to cell migration. Our finding not only provides a molecular description underlying AGS3 signalling but also demonstrates the feasibility of using GDIs to optically control Gβγ signalling independent of GPCR and Gα. The endowed precise spatial and temporal control can have a profound impact on the interrogation of Gβγ signalling in living cells and animals, exposing aberrant signalling implicated in various diseases, such as cancer and neurological diseases [64–66]. By harnessing the power of optogenetics, we are not only expanding our understanding of the signalling of a molecule that we only know very little about but also providing a methodological template for *in silico*-guided engineering of optogenetic tools for signalling control.

## Data Availability

The datasets used and/or analyzed during the current study are available from BioStudies [67]. Supplementary material is available online [68].
